# Changes in short-chain acyl-coA dehydrogenase during rat cardiac development and stress

**DOI:** 10.1111/jcmm.12541

**Published:** 2015-03-08

**Authors:** Jinxian Huang, Lipeng Xu, Qiuju Huang, Jiani Luo, Peiqing Liu, Shaorui Chen, Xi Yuan, Yao Lu, Ping Wang, Sigui Zhou

**Affiliations:** a Department of Clinical Pharmacy, GuangDong Pharmaceutical UniversityGuangzhou, China; b Institute of New Drug Research and Guangdong Province Key Laboratory of Pharmacodynamic Constituents of Traditional Chinese Medicine, Jinan University College of PharmacyGuangzhou, China; c Department of Pharmacology and Toxicology, School of Pharmaceutical Sciences, Sun Yat-sen UniversityGuangzhou, China; d Clinical Medicine Eight Years 1st Class 2007 Grade, Zhongshan School of Medicine, Sun Yat-sen UniversityGuangzhou, China; e Shenzhen Institute for Drug ControlShenzhen, China

**Keywords:** short-chain acyl-CoA dehydrogenase, peroxisome proliferator-activated receptor α, heart development, pathological cardiac hypertrophy, physiological cardiac hypertrophy

## Abstract

This study was designed to investigate the expression of short-chain acyl-CoA dehydrogenase (SCAD), a key enzyme of fatty acid β-oxidation, during rat heart development and the difference of SCAD between pathological and physiological cardiac hypertrophy. The expression of SCAD was lowest in the foetal and neonatal heart, which had time-dependent increase during normal heart development. In contrast, a significant decrease in SCAD expression was observed in different ages of spontaneously hypertensive rats (SHR). On the other hand, swim-trained rats developed physiological cardiac hypertrophy, whereas SHR developed pathological cardiac hypertrophy. The two kinds of cardiac hypertrophy exhibited divergent SCAD changes in myocardial fatty acids utilization. In addition, the expression of SCAD was significantly decreased in pathological cardiomyocyte hypertrophy, however, increased in physiological cardiomyocyte hypertrophy. SCAD siRNA treatment triggered the pathological cardiomyocyte hypertrophy, which showed that the down-regulation of SCAD expression may play an important role in pathological cardiac hypertrophy. The changes in peroxisome proliferator-activated receptor α (PPARα) was accordant with that of SCAD. Moreover, the specific PPARα ligand fenofibrate treatment increased the expression of SCAD and inhibited pathological cardiac hypertrophy. Therefore, we speculate that the down-regulated expression of SCAD in pathological cardiac hypertrophy may be responsible for ‘the recapitulation of foetal energy metabolism’. The deactivation of PPARα may result in the decrease in SCAD expression in pathological cardiac hypertrophy. Changes in SCAD are different in pathological and physiological cardiac hypertrophy, which may be used as the molecular markers of pathological and physiological cardiac hypertrophy.

## Introduction

Pressure overload to the heart, such as hypertension, results in pathological cardiac hypertrophy. Pathological cardiac hypertrophy induces a reduction in cardiac function. Furthermore, it has been reported that the progression of pathological cardiac hypertrophy results in heart failure. On the other hand, exercise training causes cardiac hypertrophy, which is defined as the athletic heart. The athletic heart is a physiological cardiac hypertrophy which is a beneficial adaptation in the cardiovascular system 1,2. As described earlier, pathological and physiological cardiac hypertrophy are associated with distinct metabolic profiles 1,3. During cardiac development, the chief cardiac energy source switches from glycolysis, during the foetal period, to fatty acid β-oxidation after birth. Substrate utilization in pathological cardiac hypertrophy resembles that of the foetal heart (decreased fatty acid oxidation, increased glycolysis), whereas physiological cardiac hypertrophy induced by exercise training is associated with enhanced fatty acid and glucose oxidation. Whether alterations in energy metabolism are a cause or consequence of cardiac hypertrophy and failure is controversial, however, there are growing bodies of evidence to suggest that switches in substrate utilization and other alterations in energy metabolism do contribute to the development of pathological cardiac hypertrophy and failure 4,5.

Fatty acids are oxidized primarily in the mitochondria *via* the β-oxidation cycle 6. Short-chain acyl-coA dehydrogenase (SCAD) is the first enzyme of the short-chain fatty acid β-oxidation spiral, which catalyses the dehydrogenation of butyryl-CoA 7. For the first time our studies have found that SCAD was obviously down-regulated in 16-week-old spontaneously hypertensive rats (SHR) by proteomics, which may partly account for the reduced fatty acid oxidation in hypertrophied hearts of SHR 8. Interestingly, studies by others have suggested that SCAD was significantly up-regulated in exercise-induced physiological cardiac hypertrophy 9. Therefore, we consider that the differences in the manner of protein expression of SCAD between pathological and physiological cardiac hypertrophy may be one of the causal factors for the difference in haemodynamic features and the prognosis of cardiac hypertrophy.

The purpose of the study was to determine whether the expression of SCAD is regulated in parallel with the known switches in myocardial energy substrate utilization during cardiac development and with the onset of stress-induced pathological and physiological cardiac hypertrophy. In this study, swim training or insulin-like growth factor-1 (IGF-1) was used as physiological stress whereas hypertension developed in SHR or phenylephrine (PE) was used as pathological stress.

## Materials and methods

### Animals and experimental protocols

For normal heart development studies, the left ventricles of foetal or neonatal wistar rats, 2-week-old juvenile wistar rats, 6-week-old young wistar rats and 16-week-old adult wistar rats (*n* = 8) were dissected out. For SHR development studies, male SHR and WKY rats aged 2, 6, 16-week-old (*n* = 8) were observed.

For pathological and physiological cardiac hypertrophy studies, male 8-week-old SHR (*n* = 8) and WKY rats (*n* = 16) were obtained. Eight WKY rats were exercised by swimming. Sedentary SHR and WKY rats (*n* = 8) were placed in the swimming apparatus for 10 min. twice a week to mimic the water stress associated with the experimental protocol 10.

To determine the effect of peroxisome proliferator-activated receptor α (PPARα) activation on pathological cardiac hypertrophy, sixteen male 8-week-old SHR were randomly divided into two groups, one group treated with saline and another group administered fenofibrate (Feno) for 8 weeks (*n* = 8). Cardiac response to the treatment was evaluated by comparison with age-matched WKY rats.

For detailed Materials and methods, please see the Data S1.

## Results

### SCAD expression and enzyme activities during wistar rats cardiac development

**Figure 1:**
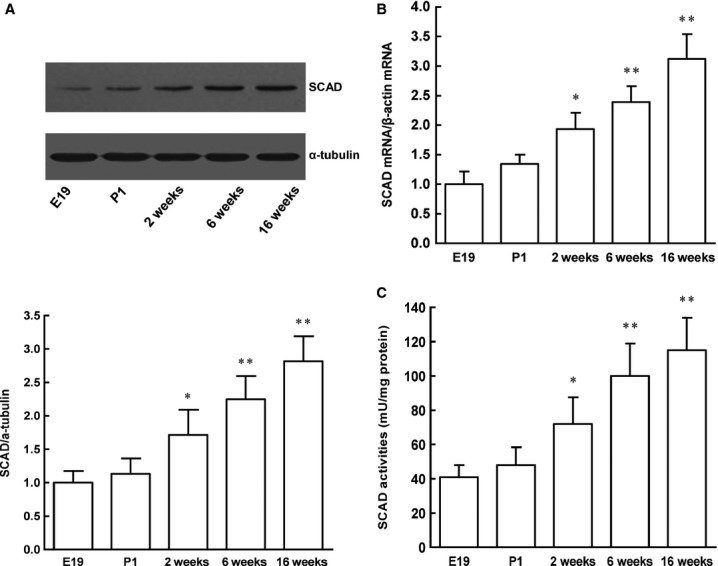
Protein and mRNA expression, enzyme activities of SCAD during the heart development in wistar rats. (A) The SCAD protein expression was significantly increased in 2, 6, 16-weeks-old wistar rats compared with foetal and neonatal wistar rats. (B) The SCAD mRNA expression was significantly increased in 2, 6, 16-weeks-old wistar rats compared with foetal and neonatal wistar rats. (C) The SCAD enzyme activities were significantly increased in 2, 6, 16-weeks-old wistar rats compared with foetal and neonatal wistar rats. E19: embryonic day 19; P1: postnatal day 1. *n* = 8 rats per group, *P < 0.05 *versus* E19, **P < 0.01 *versus* E19.

As shown in Figure1A and B, the SCAD protein and mRNA expression were lowest in the embryonic (gestational day 19) and neonatal (postnatal day 1) hearts of wistar rats, it began to increase from 2-week-old (juvenile) rats and remained to increase at 6-week-old (young) rats and 16-week-old (adult) rats. Consistent with the expression of SCAD, the enzyme activities of SCAD was significantly increased by 70.19%, 106.28% and 146.71% in 2, 6, 16-week-old wistar rats, respectively, compared with foetal and neonatal wistar rats (Fig.1C).

### SCAD expression and enzyme activities and myocardial substrate utilization during SHR cardiac development

**Figure 2:**
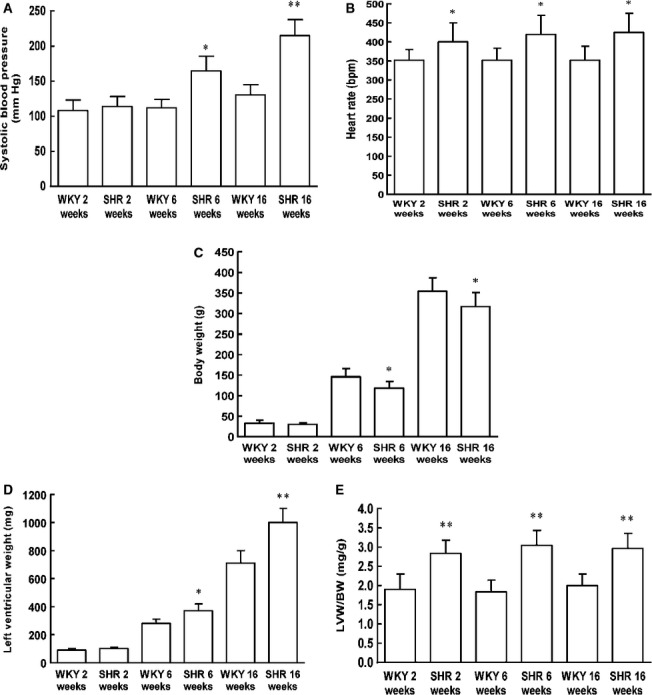
Comparison of the systolic blood pressure and the LV mass index in different ages of SHR. (A) The systolic blood pressure was significantly elevated in 6 and 16 weeks-old SHR compared with the age-matched WKY rats. (B) The heart rate was significantly increased in different ages of SHR compared with the age-matched WKY rats. (C) The body weight was significantly decreased in 6 and 16 weeks-old SHR compared with the age-matched WKY rats. (D) The LV weight was significantly elevated in 6 and 16 weeks-old SHR compared with the age-matched WKY rats. (E) The LV mass index was significantly increased in different ages of SHR compared with the age-matched WKY rats. *n* = 8 rats per group, *P < 0.05, **P < 0.01 *versus* age-matched WKY rats.

**Figure 3:**
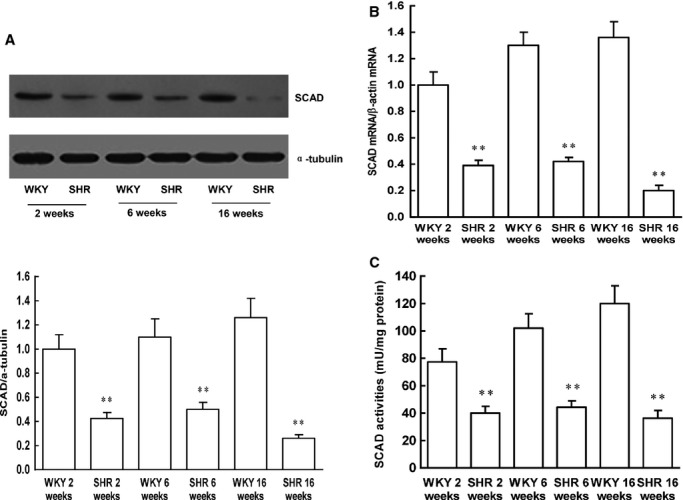
Protein and mRNA expression, enzyme activities of SCAD during the heart development in SHR. (A) The SCAD protein expression was significantly decreased in SHR compared with the age-matched WKY rats. (B) The SCAD mRNA expression was significantly decreased in SHR compared with the age-matched WKY rats. (C) The SCAD enzyme activities were significantly decreased in SHR compared with the age-matched WKY rats. *n* = 8 rats per group, **P < 0.01 *versus* age-matched WKY rats.

Despite highly significant changes in the expression of SCAD between 16-week-old SHR and age-matched WKY rats 8, we have not excluded the possibility that these could be secondary to blood pressure differences. Therefore, we further investigated whether SCAD expression was altered at early stages in SHR. Both SHR and WKY rats at 2 weeks of age had the similar systolic blood pressure. Afterwards, the systolic blood pressure was significantly elevated in SHR but not in WKY rats. Next, we analysed the LV mass index for body weight (LVW/BW) in SHR and WKY rats. The LVW/BW ratios were consistently increased in different ages of SHR (Fig.2), which suggested that cardiac hypertrophy was prior to the development of hypertension in SHR, the data were consistent with previous reports 11. A statistically significant reduction in SCAD expression and enzyme activities was observed in left ventricles at all three ages of SHR (Fig.3).

**Figure 4:**
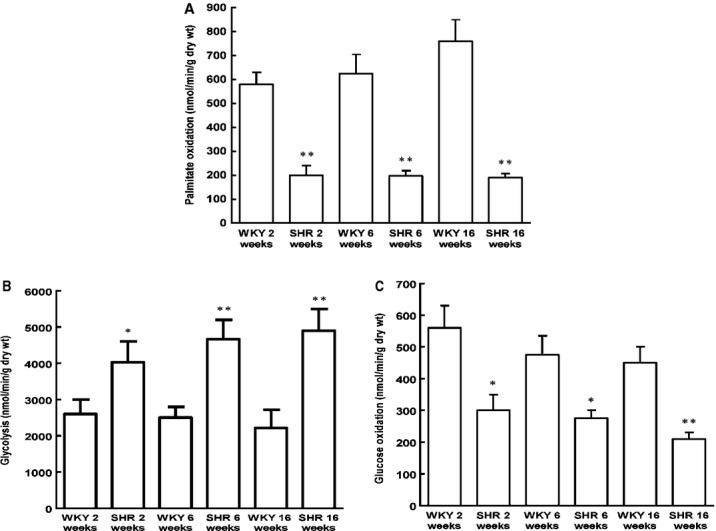
Myocardial substrate utilization during the heart development in SHR. (A) Myocardial palmitate oxidation rates were significantly decreased in SHR compared with the age-matched WKY rats. (B) Myocardial glycolysis rates were significantly increased in SHR compared with the age-matched WKY rats. (C) Myocardial glucose oxidation rates were significantly decreased in SHR compared with the age-matched WKY rats. *n* = 8 rats per group, *P < 0.05, **P < 0.01 *versus* age-matched WKY rats.

**Figure 5:**
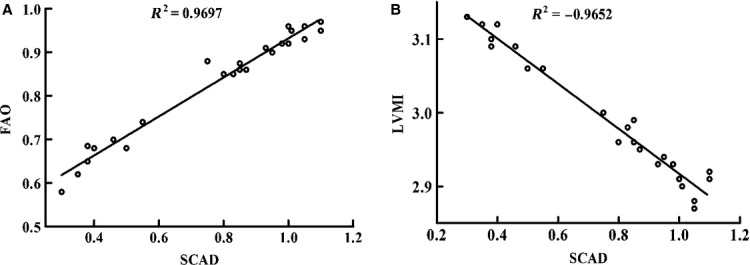
Correlation between SCAD protein expression and rates of fatty acid oxidation or LV mass index during SHR heart development. (A) A strong positive relationship was found between SCAD protein expression and rates of fatty acid oxidation during SHR heart development. (B) A strong negative relationship was found between SCAD protein expression and LV mass index during SHR heart development. FAO: fatty acid oxidation; LVMI: left ventricular mass index.

As shown in Figure4, compared with age-matched WKY rats, rates of glycolysis were significantly increased during SHR heart development. In contrast, glucose oxidation was decreased in SHR. Moreover, with palmitate as the sole exogenous fatty acid source, oxidation of palmitate, *i.e*. rates of fatty acid oxidation were significantly decreased with age. The rapid decrease in fatty acid oxidation in SHR was accordant with the decrease in SCAD expression, which suggested that the decrease in SCAD expression and enzyme activities may in part lead to the reinduction of the foetal energy metabolic programme. In addition, there was a strong positive relationship between SCAD expression and rates of fatty acid oxidation during SHR heart development. On the other hand, there was a strong negative relationship between SCAD expression and the LV mass index during SHR heart development (Fig.5).

### SCAD expression and RNA interference in pathological cardiomyocyte hypertrophy models

**Figure 6:**
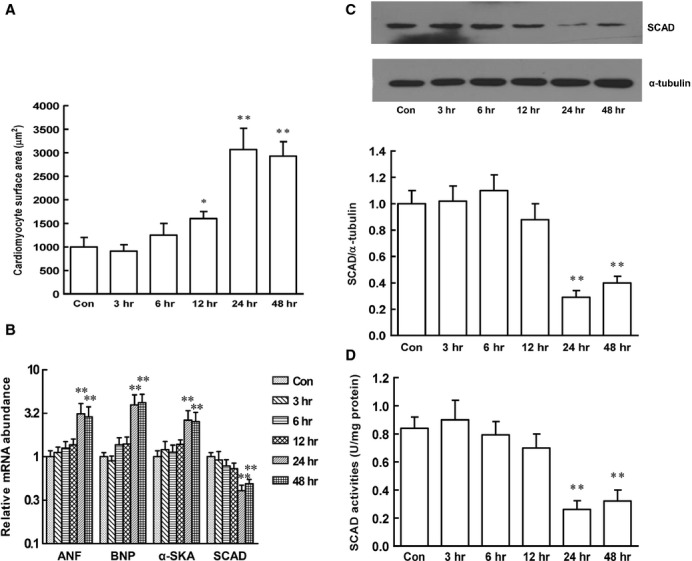
Time course of PE-induced cardiomyocytes hypertrophy on the expression and enzyme activities of SCAD in neonatal rat ventricular myocytes. (A) Cardiomyocyte surface area was significantly increased in cardiomyocytes treated with PE for 24 and 48 hrs. (B) The mRNA expression of ANF, BNP, α-SKA (marker gene of pathological hypertrophy) was significantly increased in cardiomyocytes treated with PE for 24 and 48 hrs. However, the mRNA expression of SCAD was significantly decreased in cardiomyocytes treated with PE for 24 and 48 hrs. (C) The SCAD protein expression was significantly decreased in cardiomyocytes treated with PE for 24 and 48 hrs. (D) The SCAD enzyme activities were significantly decreased in cardiomyocytes treated with PE for 24 and 48 hrs. *n* = 8 independent experiments, *P < 0.05, **P < 0.01 *versus* Con.

Next, we investigated the changes in SCAD expression and enzyme activities in pathological cardiomyocyte hypertrophy models. As shown in Figure6A and B, time-course studies found that pathological hypertrophy inducing agents PE (α-adrenergic activator) treated for 24 and 48 hrs produced a significant hypertrophic response as demonstrated by increased cardiomyocyte surface area as well as atrial natriuretic factor (ANF), brain natriuretic peptide (BNP) and α-SkA gene expression. The protein expression and enzyme activities of SCAD significantly decreased in cardiomyocytes treated with PE, which were consistent with the changes in SCAD in SHR (Fig.6C and D).

**Figure 7:**
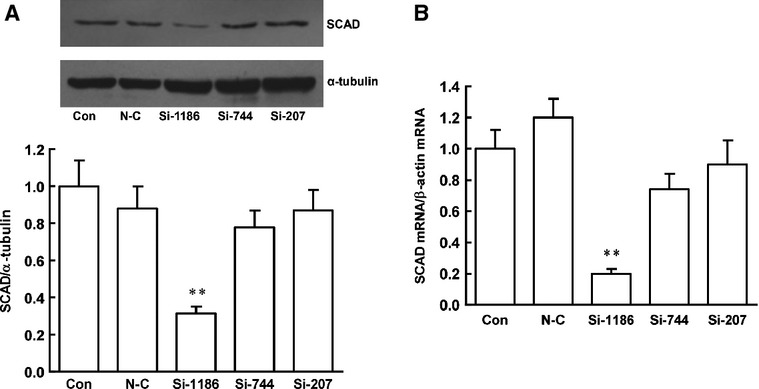
SCAD siRNA interference sequence selected results in neonatal rat ventricular myocytes. (A) siRNA1186 was the most efficient siRNA, which reduced the protein level of SCAD by 68%. (B) siRNA1186 was the most efficient siRNA, which reduced the mRNA level of SCAD by 81%. N-C: negative control siRNA. *n* = 8 independent experiments, **P < 0.01 *versus* Con.

**Figure 8:**
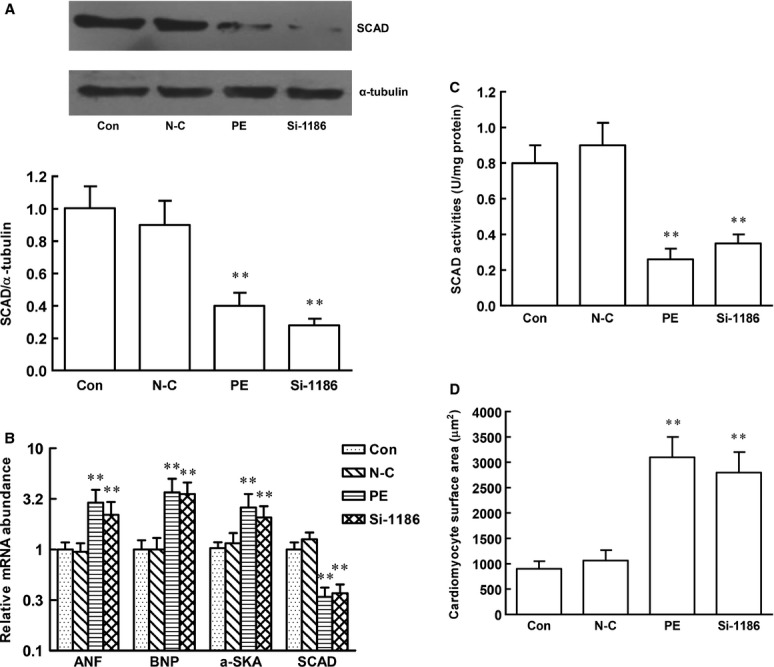
Effect of siRNA 1186 interference sequence on neonatal rat ventricular myocytes. (A) The SCAD protein expression was significantly inhibited by siRNA 1186 and PE. (B) The SCAD mRNA expression was significantly inhibited by siRNA 1186 and PE. However, the mRNA expression of ANF, BNP and α-SKA was significantly increased in cardiomyocytes treated with siRNA 1186 or PE. (C) The SCAD enzyme activities were significantly inhibited by siRNA 1186 and PE. (D) Cardiomyocyte surface area was significantly increased in cardiomyocytes treated with siRNA 1186 or PE. N-C: negative control siRNA. *n* = 8 independent experiments, **P < 0.01 *versus* Con.

To determine the effect of SCAD in pathological cardiac hypertrophy, the technique of RNA interference was employed, siRNA1186, siRNA744 and siRNA207 were transfected in cardiomyocytes for 72 hrs. Compared with negative control siRNA, siRNA1186 reduced the mRNA and protein level of SCAD by 81% and 68% respectively. (Fig.7). Therefore, siRNA 1186 was the most efficient siRNA to specifically inhibit SCAD expression and used in the subsequent experiments. Compared with the control, negative control siRNA did not affect the cardiomyocyte surface area as well as ANF, BNP and α-SkA gene expression. However, the cardiomyocyte surface area as well as ANF, BNP and α-SkA gene expression were significantly increased after siRNA 1186 treatment, which showed that SCAD siRNA treatment triggered the same pathological hypertrophy as cardiomyocytes treated with PE (Fig.8).

### Cardiac hypertrophy induced by SHR and swim training

**Figure 9:**
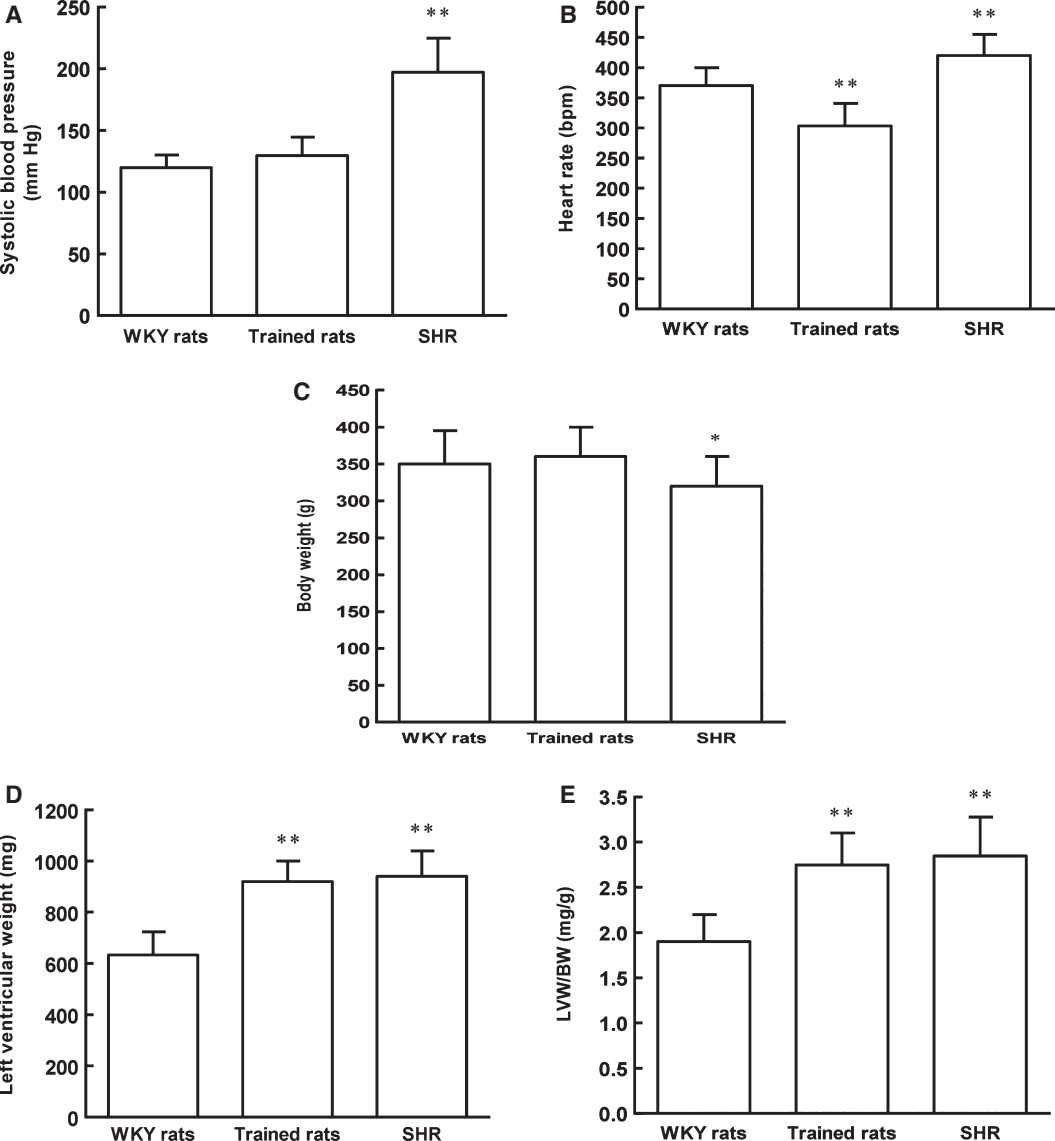
The changes in the systolic blood pressure and the LV mass index in SHR and swim-trained rats. (A) The systolic blood pressure was significantly elevated in SHR compared with the WKY and trained rats. (B) The heart rate was significantly decreased in the trained rats, however, increased in SHR compared with the WKY rats. (C) The body weight was significantly decreased in SHR compared with the WKY and trained rats. (D) The LV weight was significantly elevated in trained rats and SHR compared with the WKY rats. (E) The LV mass index was significantly increased in trained rats and SHR compared with the WKY rats. *n* = 8 rats per group, *P < 0.05, **P < 0.01 *versus* WKY rats.

As shown in Figure9, systolic blood pressure was significantly higher in the SHR group than the WKY and trained rats. There was no significant difference in systolic blood pressure between the WKY and trained rats. The LVW/BW ratios were significantly higher in SHR and trained rats than the control rats. There were no significant difference in the LVW/BW ratios between SHR and trained rats. Resting heart rate was significantly lower in the trained rats than the control rats, however, which was higher in the SHR group than the control rats.

**Figure 10:**
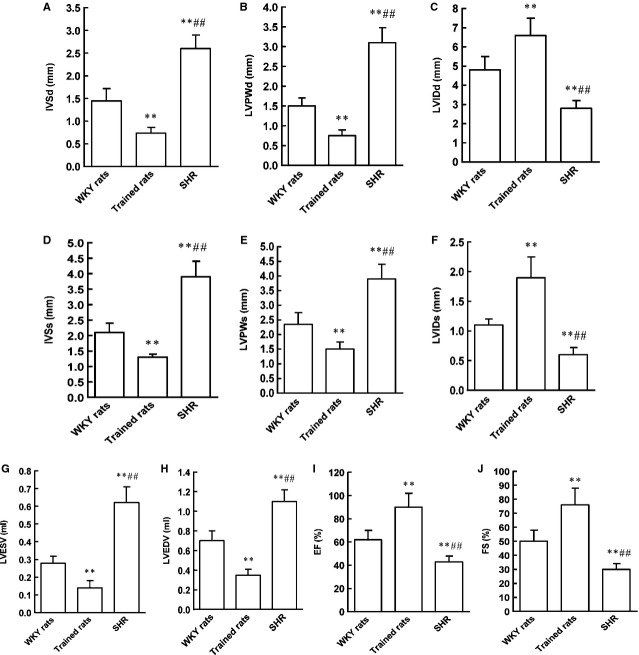
The changes in echocardiogram parameters in SHR and swim-trained rats. (A–D) The interventricular septal thickness and LV posterior wall thickness were significantly decreased at diastole and systole in trained rats compared with the control rats, however, increased at diastole and systole in SHR compared with the control rats. (E and F) The LV internal dimensions at diastole and systole were significantly increased in trained rats compared with the control rats, however, decreased at diastole and systole in SHR compared with the control rats. (G and H) The LV end volume at diastole and systole were significantly decreased in trained rats compared with the control rats, however, increased at diastole and systole in SHR compared with the control rats. (I and J) The ejection fraction and fractional shortening were significantly increased in trained rats compared with the control rats, however, decreased in SHR compared with the control rats. IVSd: interventricular septal thickness at end-diastole; LVIDd: LV internal dimensions at end-diastole; LVPWd: LV posterior wall thickness at end-diastole; IVSs: interventricular septal thickness at end-systole; LVIDs: LV internal dimensions at end-systole; LVPWs: LV posterior wall thickness at end-systole; LVESV: left ventricular end systolic volume; LVEDV: left ventricular end diastolic volume; EF: ejection fraction; FS: fractional shortening. *n* = 8 rats per group, **P < 0.01 *versus* WKY rats, ^##^*P* < 0.01 *versus* Trained rats.

To differentiate physiological and pathological cardiac hypertrophy, the dimensions of left ventricles were analysed for chamber dimensions and wall thicknesses (Fig.10). As shown in Figure1, the interventricular septal thickness and LV posterior wall thickness were significantly decreased at diastole and systole in trained rats compared with the control rats. Correspondingly, the LV internal dimensions at diastole and systole were significantly increased in trained rats, which exhibited eccentric hypertrophy. Conversely, SHR exhibited significant thickening of the interventricular septal thickness and LV posterior wall thickness at diastole and systole. Also, the LV internal dimensions at diastole and systole were significantly decreased in SHR. Subsequently, SHR exhibited markedly concentric hypertrophy. The data for ejection fraction and fractional shortening were decreased in SHR, however, increased systolic function was noted with increased ejection fraction and fractional shortening in trained rats.

**Figure 11:**
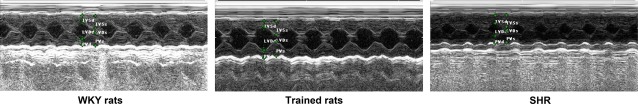
Representative chart of transthoracic M-mode echocardiograms. Note posterior wall and septal thinning and increased luminal dimension in swim-trained rats. Note the diminished luminal diameter in SHR. IVS: interventricular septum; PW: posterior wall; LVID: left ventricular internal dimensions.

### Changes in SCAD and PPARα expression differ between pathological and physiological cardiac hypertrophy *in vivo* and *in vitro*

To investigate the possible mechanisms for the decrease in SCAD expression in pathological cardiac hypertrophy, the expression of PPARα was determined. PPARα is a critical transcriptional regulator of fatty acid oxidation. As depicted in Figure2A and B, the protein and mRNA expression of SCAD and PPARα were markedly reduced in SHR compared with WKY rats, whereas the expression of SCAD and PPARα was significantly increased in trained rats. Enzyme activities of SCAD had the opposite trends between SHR and trained rats (Fig.2C). As shown in Figure3, compared with WKY rats, SHR induced a shift in myocardial substrate utilization, as indicated by decrease in fatty acid oxidation and increase in glycolysis. However, the rate of fatty acid and glucose oxidation was significantly increased in trained rats. The changes in fatty acid oxidation between SHR and exercise training rats were accordant with the changes in SCAD expression.

**Figure 12:**
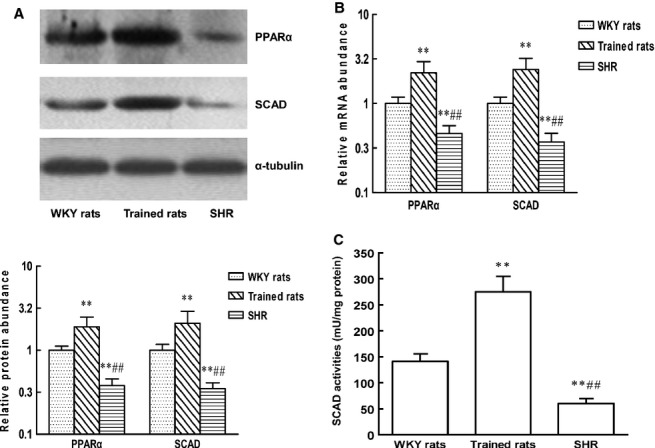
The expression of PPARα and SCAD, the enzyme activities of SCAD in SHR and swim-trained rats. (A) The protein expression of PPARα and SCAD was significantly decreased in SHR, however, increased in swim-trained rats. (B) The mRNA expression of PPARα and SCAD was significantly decreased in SHR, however, increased in swim-trained rats. (C) The enzyme activities of SCAD were significantly decreased in SHR, however, increased in swim-trained rats. *n* = 8 rats per group, **P < 0.01 *versus* WKY rats, ^##^*P* < 0.01 *versus* Trained rats.

**Figure 13:**
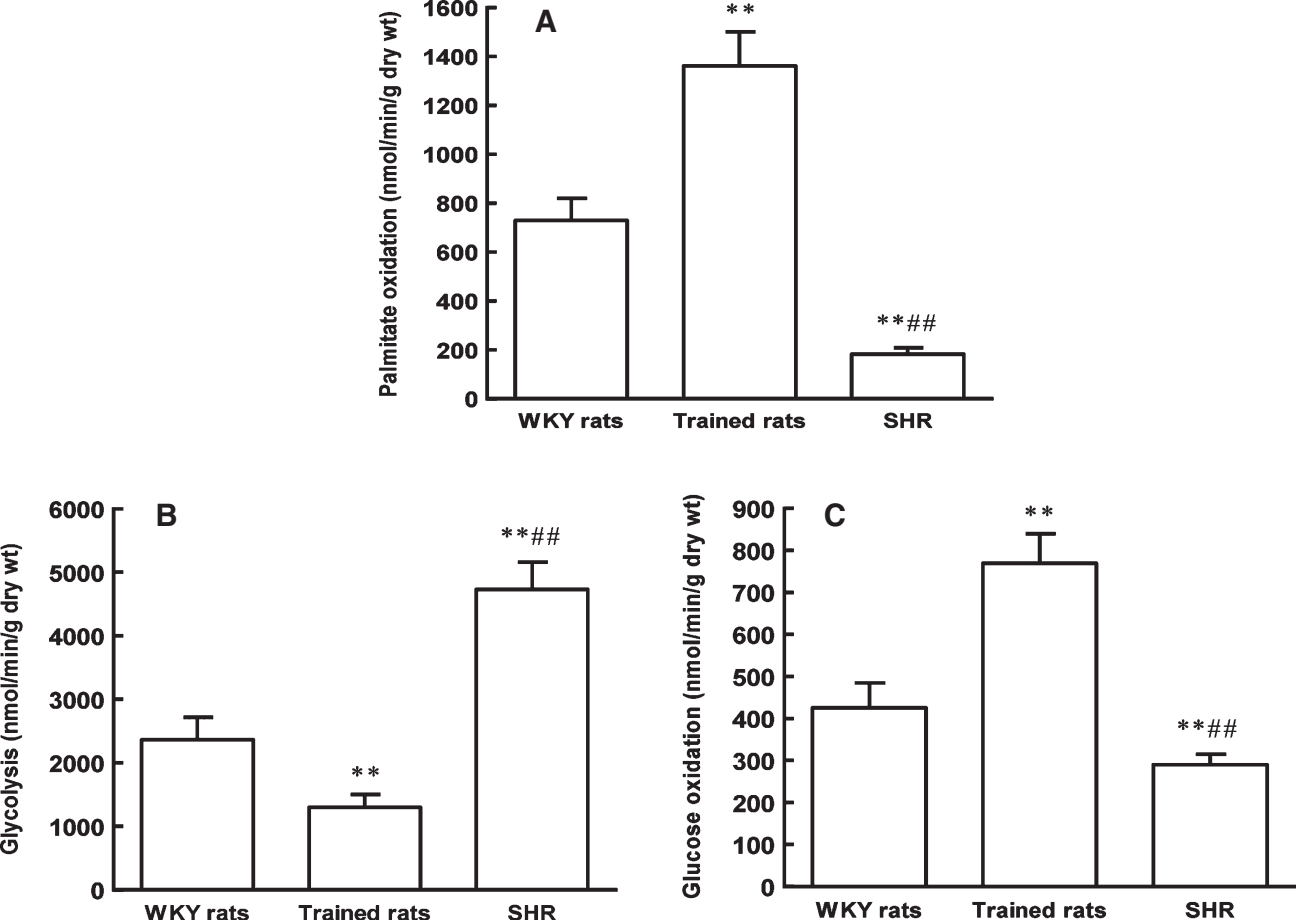
Myocardial substrate utilization in SHR and swim-trained rats. (A) Myocardial palmitate oxidation rates were significantly decreased in SHR, however, increased in swim-trained rats. (B) Myocardial glycolysis rates were significantly increased in SHR, however, decreased in swim-trained rats. (C) Myocardial glucose oxidation rates were significantly decreased in SHR, however, increased in swim-trained rats. *n* = 8 rats per group, **P < 0.01 *versus* WKY rats, ^##^*P* < 0.01 *versus* Trained rats.

We also investigated the changes in SCAD and PPARα expression between pathological and physiological cardiac hypertrophy *in vitro*. Cardiomyocytes were treated with either pathological hypertrophic agonist PE, or physiological hypertrophic agonist IGF-1. Cardiomyocyte surface area was significantly increased in the two models (Fig.4A). Cardiomyocytes treated with PE showed the expected increase in ANF, BNP and α-SkA (a marker of pathological hypertrophy) mRNA levels consistent with pathological cardiomyocytes hypertrophic growth. However, IGF-1 induced the expression of both ANF and BNP but without a change in α-SkA levels, which was consistent with the induction of physiological cardiomyocytes hypertrophy 12. Consistent with the changes in rats, SCAD and PPARα protein and mRNA levels were markedly reduced in cardiomyocytes treated with PE, whereas the expression of SCAD and PPARα was significantly increased in cardiomyocytes treated with IGF-1(Fig.4 B–D).

**Figure 14:**
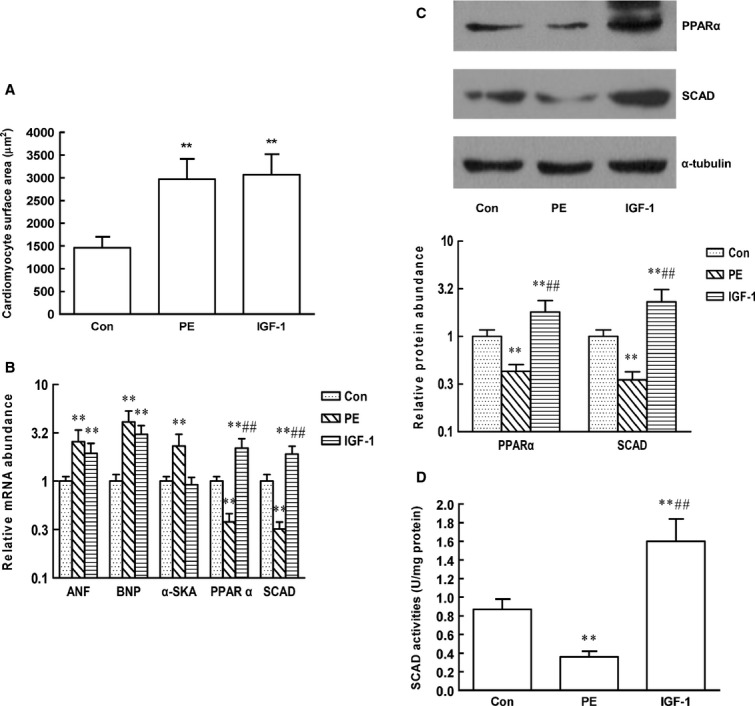
Effect of PE and IGF-1 on the expression of PPARα and SCAD, the enzyme activities of SCAD in neonatal rat ventricular myocytes. (A) Cardiomyocyte surface area was significantly increased in cardiomyocyte treated with PE and IGF-1. (B) SCAD and PPARα mRNA levels were markedly reduced in cardiomyocytes treated with PE, whereas increased in cardiomyocytes treated with IGF-1. The mRNA levels of ANF, BNP were both increased in cardiomyocytes treated with PE and IGF-1. However, the mRNA expression of α-SkA was only increased in cardiomyocytes treated with PE, which is a marker of pathological hypertrophy. (C) SCAD and PPARα protein levels were markedly reduced in cardiomyocytes treated with PE, whereas increased in cardiomyocytes treated with IGF-1. (D) SCAD enzyme activities were markedly reduced in cardiomyocytes treated with PE, whereas increased in cardiomyocytes treated with IGF-1. *n* = 8 independent experiments, **P < 0.01 *versus* Con, ^##^*P* < 0.01 *versus* PE.

### Fenofibrate activates PPARα and SCAD to inhibit pathological cardiac hypertrophy *in vivo* and *in vitro*

Consistent with the changes in SCAD expression, the protein and mRNA levels of PPARα were decreased in SHR (Fig.5A and B). To determine the effect of PPARα activation on the expression of SCAD and cardiac hypertrophy, SHR were treated with fenofibrate (Feno, the PPARα agonist) for 8 weeks. The LVW/BW ratios and systolic blood pressure were significantly increased in SHR compared with WKY rats. Treatment with Feno significantly reduced the LVW/BW ratios compared to those in SHR without Feno. However, the systolic blood pressure was not significantly affected by treatment with Feno (Fig.6). Feno significantly increased the expression and enzyme activities of SCAD in SHR (Fig.5C). In addition, rates of glycolysis were significantly decreased and rates of fatty acid oxidation were significantly increased in SHR treated with Feno (Fig.7).

**Figure 15:**
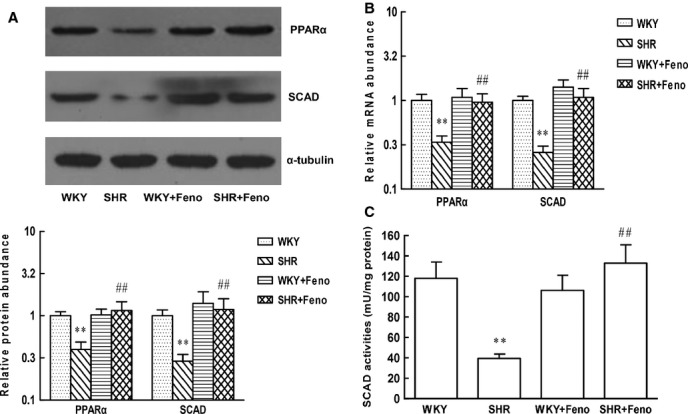
The changes in protein and mRNA expression, enzyme activities of SCAD in SHR treated with Feno. (A) The PPARα and SCAD protein expression was significantly decreased in SHR, however, increased in SHR pre-treated with Feno. (B) The PPARα and SCAD mRNA expression was significantly decreased in SHR, however, increased in SHR pre-treated with Feno. (C) The SCAD enzyme activities were significantly decreased in SHR, however, increased in SHR pre-treated with Feno. *n* = 8 rats per group, **P < 0.01 *versus* WKY rats, ^##^*P* < 0.01 *versus* SHR.

**Figure 16:**
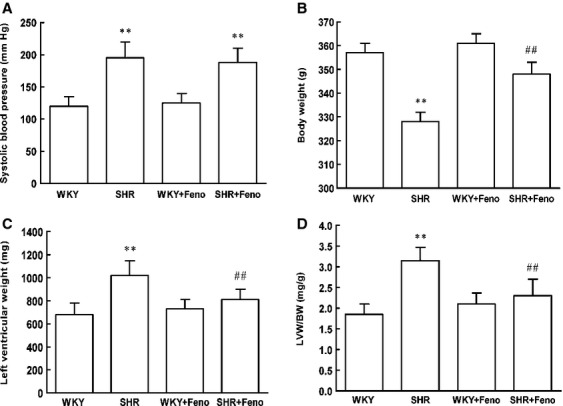
Comparison of the systolic blood pressure and the LV mass index in SHR treated with or without Feno. (A) The systolic blood pressure was significantly elevated in SHR compared with the WKY rats, and Feno had no effect on the systolic blood pressure in SHR. (B) The body weight was significantly decreased in SHR compared with the WKY rats, and Feno increased the body weight in SHR. (C) The LV weight was significantly elevated in SHR compared with the WKY rats, and Feno decreased the LV weight in SHR. (D) The LV mass index was significantly increased in SHR compared with the WKY rats, and Feno decreased the LV mass index in SHR. *n* = 8 rats per group, **P < 0.01 *versus* WKY rats, ^##^*P* < 0.01 *versus* SHR.

**Figure 17:**
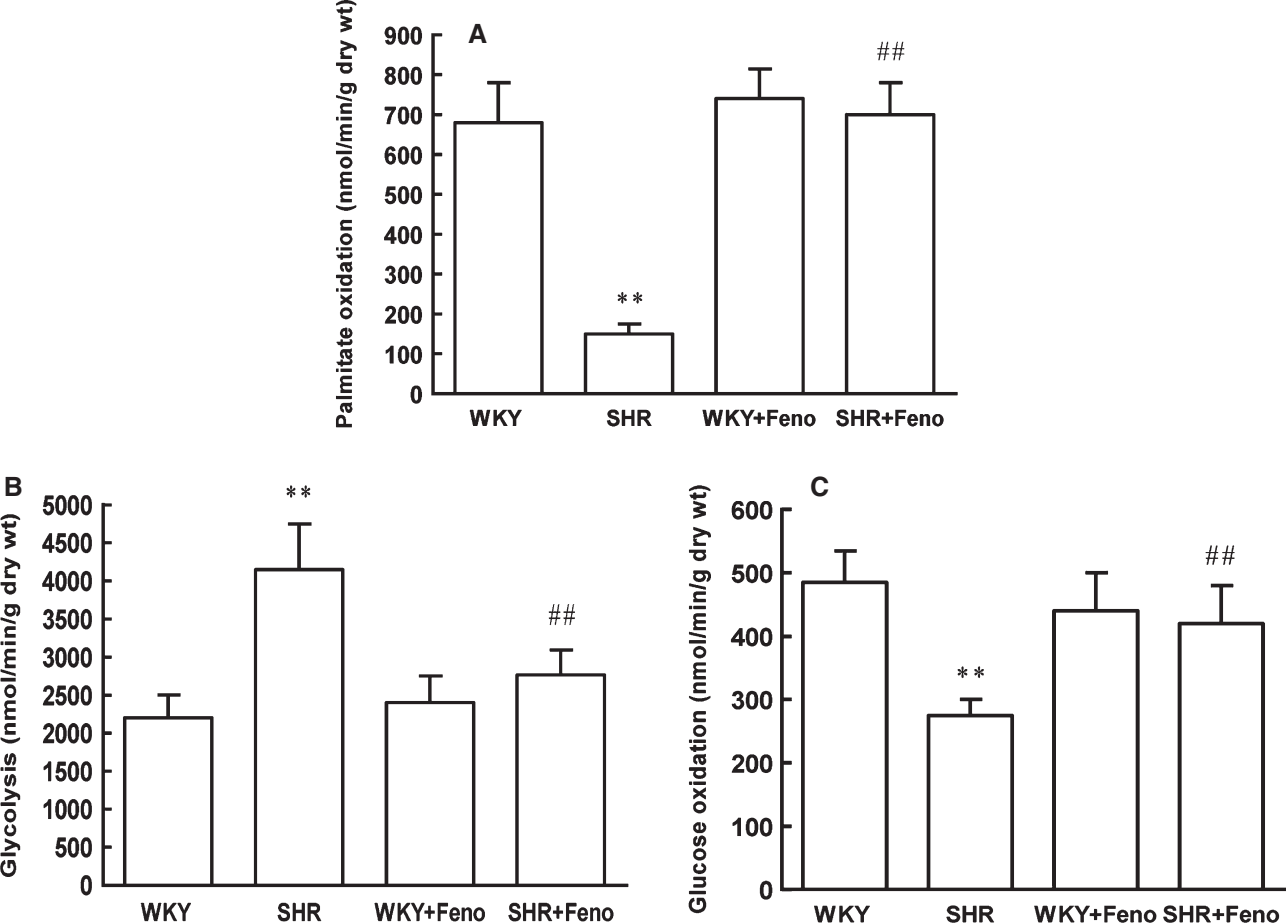
Myocardial substrate utilization in SHR treated with Feno. (A) Myocardial palmitate oxidation rates were significantly decreased in SHR, however, increased in SHR pre-treated with Feno. (B) Myocardial glycolysis rates were significantly increased in SHR, however, decreased in SHR pre-treated with Feno. (C) Myocardial glucose oxidation rates were significantly decreased in SHR, however, increased in SHR pre-treated with Feno. *n* = 8 rats per group, **P < 0.01 *versus* WKY rats, ^##^*P* < 0.01 *versus* SHR.

We also investigated the effect of PPARα activation on pathological cardiomyocyte hypertrophy models. Cardiomyocytes were pre-treated with Feno and subsequently with PE for 48 hrs (Fig.8) or SCAD siRNA for 72 hrs (Fig.9). Compared with control cardiomyocytes, PE or SCAD siRNA treatment significantly decreased the expression and enzyme activities of SCAD, increased cardiomyocyte surface area as well as ANF, BNP and α-SkA gene expression, which showed that PE and SCAD siRNA treatment triggered the same pathological cardiomyocytes hypertrophy. Treatment with Feno significantly increased the expression and enzyme activities of SCAD compared to those in hypertrophic cardiomyocyte without Feno. In addition, cardiomyocyte surface area, ANF, BNP and α-SkA gene expression were significantly decreased in hypertrophic cardiomyocyte pre-treated with Feno. These data demonstrated that activation of PPARα and SCAD suppressed pathological cardiac hypertrophy *in vivo* and *in vitro*.

**Figure 18:**
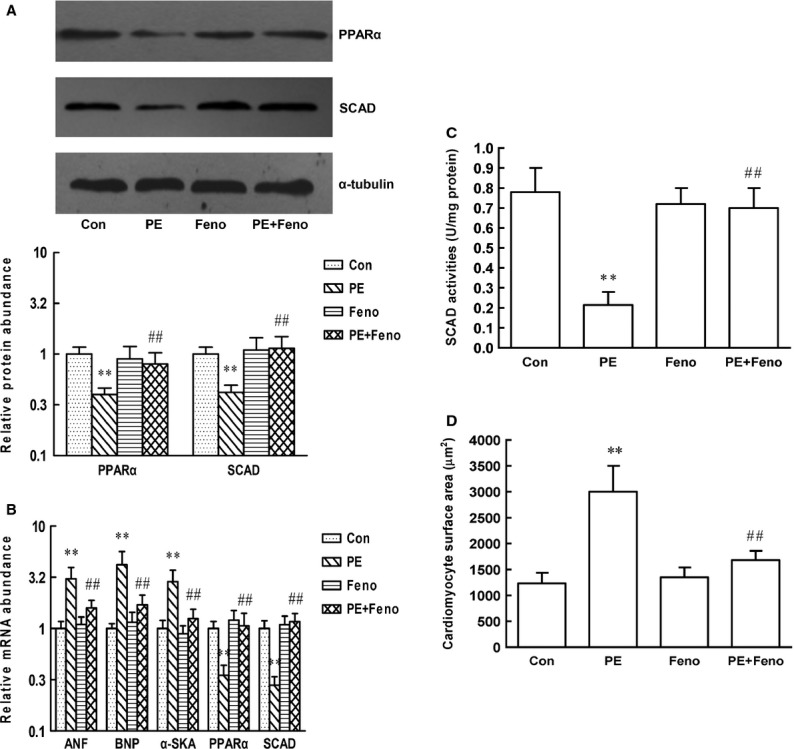
Effect of Feno on the expression and enzyme activities of SCAD, the parameters of cardiomyocyte hypertrophy in neonatal rat ventricular myocytes treated with PE. (A) The PPARα and SCAD protein expression was significantly decreased in cardiomyocytes treated with PE, however, increased in cardiomyocytes pre-treated with Feno. (B) The PPARα and SCAD mRNA expression was significantly decreased in cardiomyocytes treated with PE, however, increased in cardiomyocytes pre-treated with Feno. The mRNA expression of ANF, BNP and α-SKA was significantly increased in cardiomyocytes treated with PE, however, decreased in cardiomyocytes pre-treated with Feno. (C) The SCAD enzyme activities were significantly decreased in cardiomyocytes treated with PE, however, increased in cardiomyocytes pre-treated with Feno. (D) The cardiomyocyte surface area was significantly increased in cardiomyocytes treated with PE, however, decreased in cardiomyocytes pre-treated with Feno. *n* = 8 independent experiments, **P < 0.01 *versus* Con, ^##^*P* < 0.01 *versus* PE.

**Figure 19:**
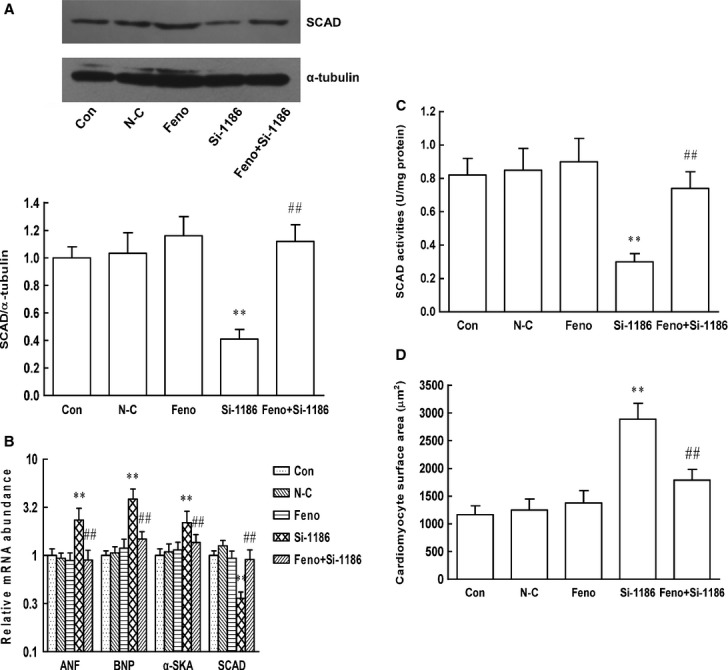
Effect of Feno on the expression and enzyme activities of SCAD, the parameters of cardiomyocyte hypertrophy in neonatal rat ventricular myocytes treated with siRNA 1186 interference sequence. (A) The PPARα and SCAD protein expression was significantly decreased in cardiomyocytes treated with siRNA 1186, however, increased in cardiomyocytes pre-treated with Feno. (B) The PPARα and SCAD mRNA expression was significantly decreased in cardiomyocytes treated with siRNA 1186, however, increased in cardiomyocytes pre-treated with Feno. The mRNA expression of ANF, BNP and α-SKA was significantly increased in cardiomyocytes treated with siRNA 1186, however, decreased in cardiomyocytes pre-treated with Feno. (C) The SCAD enzyme activities were significantly decreased in cardiomyocytes treated with siRNA 1186, however, increased in cardiomyocytes pre-treated with Feno. (D) The cardiomyocyte surface area was significantly increased in cardiomyocytes treated with siRNA 1186, however, decreased in cardiomyocytes pre-treated with Feno. N-C: negative control siRNA. *n* = 8 independent experiments, **P < 0.01 *versus* Con, ^##^*P* < 0.01 *versus* si-1186.

## Discussion

For the first time we show that SCAD expression and enzyme activities gradually increased from embryonic stage to adulthood. On the contrary, SCAD expression and enzyme activities significantly decreased in pathological cardiac hypertrophy *in vivo* and *in vitro*. The present data have also shown that pathological and physiological cardiac hypertrophy exhibited divergent SCAD changes in myocardial fatty acids utilization.

### SCAD reactivation of the foetal metabolic gene regulatory programmes in pathological cardiac hypertrophy

Numerous investigators have explored the regulation of metabolic enzyme encoding genes in cardiac hypertrophy and heart failure 13,14. The changes in fatty acid oxidation enzyme encoding genes, including the medium chain acyl-coA dehydrogenase gene, were characterized in the heart 15. However, the changes in SCAD during rat cardiac development and stress remain unclear.

SCAD expression and enzyme activities were lowest in hearts during the foetal and neonatal stages of the developing rats when glucose serves as the chief energy substrate. From juvenile to adult heart, the expression and enzyme activities of SCAD increased markedly in parallel with the transition from reliance on glycolysis to fatty acids β-oxidation as the major energy source, which demonstrated that the expression patterns of SCAD genes were paralleled the known developmental energy substrate switch from glucose to fatty acids.

Interestingly, SCAD expression and enzyme activities during SHR development were consistent with the embryonic and neonatal hearts of wistar rats. In this study, we observed a significant decrease in cardiac SCAD expression and enzyme activities in 2- (pre-hypertensive stage), 6- (developing hypertension) and 16- (established hypertension) week-old SHR compared with corresponding WKY rats, which suggested that reduction in SCAD expression may contribute directly to the pathogenesis of cardiac hypertrophy and was not an adaptive response caused by long-term differences in blood pressure. Compared with age-matched WKY rats, rates of glycolysis were significantly increased in SHR. On the contrary, rates of fatty acid oxidation were significantly decreased with age in SHR. Therefore, the pattern for SCAD expression during SHR development was consistent with the embryonic and neonatal hearts of normal rats, which suggested that the decrease in SCAD expression may in part lead to the recapitulation of the foetal energy metabolic gene regulatory programme in SHR.

In this study, SCAD expression and enzyme activities in PE-induced pathological cardiomyocyte hypertrophy models were also decreased, which were accordant with the changes in SCAD in SHR. Moreover, SCAD siRNA treatment triggered the same pathological hypertrophy as cardiomyocytes treated with PE, which showed that the down-regulation of SCAD expression played an important role in pathological cardiac hypertrophy.

### Pathological and physiological cardiac hypertrophy exhibit different expression pattern of SCAD

In this study, the swim-trained rats exhibited physiological cardiac hypertrophy and SHR exhibited pathological cardiac hypertrophy. SCAD expression and enzyme activities were markedly reduced in SHR, whereas increased in swimming training rats, despite a similar degree of cardiac hypertrophy. This alteration further confirmed the previous observations by proteomics 8,9. In this research, SHR induced a shift in myocardial substrate utilization, fatty acid oxidation was decreased and glycolysis was increased. However, the rates of fatty acid and glucose oxidation were significantly increased in trained rats. The down-regulation of SCAD expression in pathological cardiac hypertrophy was consistent with the known decrease in fatty acid oxidation in response to hypertension. On the other hand, up-regulation of SCAD expression in physiological models was consistent with the known increase in fatty acid oxidation in response to exercise.

Meanwhile, SCAD expression and enzyme activities in PE-induced pathological cardiomyocyte hypertrophy were decreased. However, IGF-1 increased SCAD expression and enzyme activities in physiological cardiomyocyte hypertrophy. Taken together, these studies suggested that pathological and physiological cardiac hypertrophy exhibited divergent SCAD changes in myocardial fatty acids utilization.

The principal transcriptional regulator of fatty acid oxidation enzyme genes is PPARα, a member of the ligand activated nuclear receptor superfamily 16. PPARα expression was elevated in hearts of trained rats and down-regulated in several models of pathological cardiac hypertrophy, concomitant with the switch from fatty acid to glucose utilization 17–19. PPARα knockout mice have markedly reduced fatty acid oxidation and exhibited cardiac lipid accumulation and pathological cardiac hypertrophy 20. In addition, activation of PPARα inhibited pathological cardiac hypertrophy *via* various signalling pathways 21,22. Therefore, PPARα may be the mediator, at least in part, of the energy metabolic substrate switch from fatty acid oxidation to glycolysis during pathological cardiac hypertrophy. Previous studies have also shown that constitutive expression level of SCAD was much lower in PPARα-null mice than wild-type mice, which demonstrated that the expression of SCAD gene may be regulated by PPARα 23.

In this study, the expression of PPARα was decreased in SHR and PE-induced pathological cardiomyocyte hypertrophy. In contrast, swim training and IGF-1 induced physiological cardiac hypertrophy resulted in up-regulation of PPARα, the trends were accordant with the changes in SCAD, which demonstrated that PPARα may serve a critical role in different SCAD changes between pathological and physiological cardiac hypertrophy.

We further determine the effect of PPARα activation on the expression of SCAD and pathological cardiac hypertrophy *in vivo* and *in vitro*. The specific PPARα ligand Feno significantly reduced the LVW/BW ratios and increased the expression of SCAD and fatty acid oxidation in SHR. Moreover, Feno significantly increased the expression and enzyme activities of SCAD, inhibited pathological cardiomyocyte hypertrophy induced by PE or SCAD siRNA. These data demonstrated that the deactivation of PPARα may result in the decrease in SCAD expression in pathological cardiac hypertrophy.

In conclusion, we have shown that the expression of SCAD was coordinately repressed in pathological cardiac hypertrophy: a recapitulation of the foetal energy metabolic gene regulatory programme. Interestingly, pathological and physiological cardiac hypertrophy exhibited divergent SCAD changes in myocardial fatty acids utilization. We propose that this regulatory pathway represents a useful target for future experimental studies aimed at the characterization of alterations about fatty acids metabolism in cardiac hypertrophy. An alternate strategy to directly inhibiting pathological heart growth is to activate SCAD regulators of physiological heart growth.
