# Supplementary material for: Changes in short-chain acyl-coA dehydrogenase during rat cardiac development and stress
Source: J Cell Mol Med. 2015 Mar 8;19(7):1672–88. doi: 10.1111/jcmm.12541 (PMC4511364; doi:10.1111/jcmm.12541)
Supplement: Supplementary file 1 [file jcmm0019-1672-sd1.doc]

**Materials and methods**

**Animals and experimental protocols**

The investigation conforms with the Guide for the Care and Use of Laboratory Animals published by the US National Institutes of Health (NIH Publication, 8th Edition, 2011). The research protocol is approved by the Ethical Committee for Animal Experiments of GuangDong Pharmaceutical University. SHR and Wistar-Kyoto (WKY) rats were obtained from Slac Laboratory Animal Limited Company (Shanghai, China). Wistar rats were obtained from [Guangdong Medical Laboratory Animal Center](http://www.gdmlac.com.cn/index.php?q=en) (Guangzhou, China).

Male and female wistar rats (225–250 g) were housed under standard conditions with a 12-hour light/12-hour dark cycle and free access to water and chow. Two females were placed with a male overnight and examined the next morning for the presence of sperm in a vaginal smear. The day on which sperm were found was designated as day 0 of gestation, and the females were caged individually thereafter.

For heart development studies, pregnant rats from day 19 of gestation were anesthetized with sodium pentobarbital (45 mg/kg body weight by ip) to obtain fetal rats by cesarean section. The fetal rats (n=32) were then killed by decapitation, and whole hearts were removed. Similarly at day 1 after birth, offspring (n=32) were then killed by cervical dislocation, and individual whole hearts were collected. Using a dissecting microscope, the left ventricles of fetal or neonatal rats were dissected out. 2-week-old juvenile rats, 6-week-old young rats and 16-week-old adult rats (n=8) were anesthetized with sodium pentobarbital (45 mg/kg body weight by ip), the chest cavity was opened, and individual whole hearts were collected and the left ventricles were isolated. All the rats were killed by removing the hearts. In addition, for SHR development studies, male SHR and WKY rats aged 2, 6, 16-week-old (n=8) were obtained from Slac Laboratory Animal Limited Company (Shanghai, China). The left ventricles were snap frozen in liquid N_2_, and stored at -80ºC. To extract sufficient mRNA and protein, four fetal or neonatal rat left ventricles were pooled in age-matched groups, and at least eight groups of pooled samples were used from each age point for studies. SCAD enzyme activity was measured with a commercially available kit from Shanghai Genmed Gene Pharmaceutical Technology Limited Company (Shanghai, China).

For pathological and physiological cardiac hypertrophy studies, male 8-week-old SHR (n=8) and WKY rats (n=16) were obtained. Eight WKY rats were exercised by swimming. The training sessions were performed during the rats’ dark cycle and consisted of 60-min swimming sessions five days/week for 8 weeks in a swimming apparatus containing warmed water (30-32ºC). Exercise duration was increased gradually until rats could swim for 60 min. Sedentary SHR and WKY rats (n=8) were placed in the swimming apparatus for 10 min twice a week to mimic the water stress associated with the experimental protocol [1].

To determine the effect of PPARα activation on pathological cardiac hypertrophy, sixteen male 8-week-old SHR were randomly divided into two groups, one group treated with 1% carboxymethyl cellulose and another administered fenofibrate (Feno) orally by gavage at a dose of 100 mg/kg body weight for 8 weeks (n=8). Cardiac response to the treatment was evaluated by comparison with age-matched WKY rats. Heart rate and systolic blood pressure by the tail-cuff method were recorded weekly. At the end of 8 weeks protocol animals were anesthetized with sodium pentobarbital (45 mg/kg body weight by ip), the hearts removed and the left ventricle with the septum weighed and normalized by body weight to determine cardiac hypertrophy.

**Echocardiographic examination**

At the end of the experiment, rats were anesthetized with sodium pentobarbital (45 mg/kg body weight by ip). Two-dimensionally guided M-mode echocardiography was performed using a Technos MPX ultrasound system (ESAOTE, Italy) equipped with an 8.5-MHz imaging transducer as described previously [2].

**Cardiomyocyte isolation and culturing**

Neonatal rat ventricular myocytes were isolated from the cardiac ventricles of one- to three-day-old wistar rats as previously described [3]. The animals were decapitated and the hearts were rapidly excised from the thoracic cavity and ventricular tissue fragments were repeatedly treated with 2 mg/ml trypsin to release the cells. To remove noncardiomyocytes, isolated cells were preplated in cell culture dishes in 50 ml DMEM with 5% FCS for 45 min. During this period, most noncardiomyocytes (mainly fibroblasts) attached to the dish, whereas cardiomyocytes remained in solution. The cardiomyocytes were subsequently transferred to separate tissue culture dishes and allowed to attach. Cardiomyocytes were cultured at 37°C under 5% CO_2_ in DMEM and supplemented with 10% FCS and penicillin streptomycin (100 IU/ml and 100 μg/ml, respectively). For experiments cardiomyocytes were first serum starved for 24 h, then treated with 20 μmol/l PE or 20 μmol/l IGF-1 for 24 h with or without 10 μmol/l Feno.

**Transfection of cardiomyocyte with SCAD siRNA**

SCAD siRNA were obtained from Shanghai GenePharma Limited Company (Shanghai, China). The following sequences were used: siRNA1186 sense 5´-CCGCAUCACUGAGAUCAUTT-3´ and anti-sense 5´-AUAGAUCUCAGUGAU GCGGTT-3´; siRNA744 sense 5´-CAGCUAACCUCUUUGATT-3´ and anti-sense 5´-UCAAAGAUGAGGUUAGCUGTT-3´; siRNA207 sense 5´-CCAGCUGGACAA GGAACAUTT-3´ and anti-sense 5´-AUGUUCCUUGUCCAGCUGGTT-3´. A universal scrambled negative control siRNA (Shanghai GenePharma Limited Company) was used for control studies.

Cardiomyocytes were cultured for 24 h in complete medium then changed to 5% FBS containing medium for another 24 h. Cells in 5% medium were then maintained in serum free medium in the presence or absence of SCAD siRNA (20 μM) for 72h using Lipofectamine 2000 as transfecting reagent from Invitrogen. The transfected cells were pretreated with 10 μmol/L Feno for 24 h. The silencing efficacy was confirmed by RT-PCR and Western blotting after transfection.

**Substrate metabolism in isolated working rat hearts**

All hearts were prepared and perfused in the working mode, using protocols that have been previously described [4]. In brief, the working heart buffer was Krebs-Henseleit buffer containing 118.5 mM NaCl, 25 mM NaHCO_3_, 4.7 mM KCl, 1.2 mM MgSO_4_, 1.2 mM KH_2_PO_4_, 2.5 mM CaCl_2_, 0.5 mM EDTA, and 5 mM glucose, gassed with 95% O_2_-5% CO_2_ and supplemented with 0.4 mM palmitate bound to 3% BSA in the absence of insulin. Glycolytic flux was determined by measuring the amount of ^3^H_2_O released from the metabolism of exogenous [5-^3^H]glucose (specific activity, 177 Gbq/mol). Glucose oxidation was determined by trapping and measuring ^14^CO_2_ released by the metabolism of [U-^14^C]glucose (specific activity, 96 Mbq/mol). Palmitate oxidation was determined in separate perfused hearts by measuring the amount of ^3^H_2_O released from [9,10-^3^H] palmitate (specific activity, 42 Gbq/mol).

**Real-time PCR assay**

Total RNA was isolated using TRIzol reagent (Invitrogen) and converted to cDNA using MLV reverse transcriptase (Promega). Quantitative real-time PCR analyses were performed using SYBR Green PCR Master Mix kit with a MyiQ real-time PCR thermal cycler (Bio-Rad). Standard curves were generated using β-actin primers. The mRNA expression levels of test genes were normalized to β-actin levels. Primers were synthesized by Shanghai Sangon Biotech Limited Company (Shanghai, China). The primers used were as follows: PPARα, sense 5'-CCTGGCAATGCACTGAAC ATC-3' and anti-sense 5'-ACGCCGTTGGCTACCATCTTG-3'; SCAD, sense 5'-TGCCCTATGTTTCGCACCTC-3' and anti-sense 5'-TTCAATGCCCATCAT CCCTT-3'; atrial natriuretic factor (ANF), sense 5'-GGAAGTCAACCCGTCTCA-3' and anti-sense 5'-AGCCCTCAGTTTGCTTTT-3'; brain natriuretic peptide (BNP), sense 5'-TTTGGGCAGAAGATAGACCG-3' and anti-sense 5'-AGAAGAGCCGCA GGCAGAG-3'; alpha skeletal actinin (α-SkA), sense 5'-CAGGCGGTGCTGTCT CTCTAT-3' and anti- sense 5'-GGCAGGGCATAACCCTCATA-3'; β-actin, sense 5'-GGACTTCGAGCAAGAGATGG-3' and anti-sense 5'-AGCACTGTGTTGGCG TACAG-3'.

**Western Blotting assay**

The protein samples were run on 12% SDS-PAGE gels under reducing conditions and transferred onto PVDF membranes. The membranes were blocked with 5% nonfat milk in Tris-buffered saline Tween 20 for 2 h at room temperature and then probed with antibodies. Primary antibodies involved in this study included SCAD (ab156571, Abcam) diluted at 1:1000, PPARα (P0869, Sigma) diluted at 1:1000 and α-tubulin (T6074, Sigma) diluted at 1:2000. After incubation with corresponding secondary antibodies, the bands were detected using an ECL kit (Pierce) and visualized and quantified using a Bio-Rad Image Analyzer densitometry system.

**Statistical analysis**

Data are presented as mean ± SEM. Statistical evaluation of the data was performed by one-way analysis of variance followed by a Bonferroni-corrected Student’s t test for multiple comparisons. Correlation of SCAD expression with rates of fatty acid oxidation and the LVW/BW ratios was determined using Spearman’s rank correlation coefficient. A P-value of < 0.05 was considered significant.

**References**

[1] [**Garciarena CD**](http://www.ncbi.nlm.nih.gov/pubmed?term=Garciarena CD%5BAuthor%5D&cauthor=true&cauthor_uid=19221208)**,** [**Pinilla OA**](http://www.ncbi.nlm.nih.gov/pubmed?term=Pinilla OA%5BAuthor%5D&cauthor=true&cauthor_uid=19221208)**,** [**Nolly MB**](http://www.ncbi.nlm.nih.gov/pubmed?term=Nolly MB%5BAuthor%5D&cauthor=true&cauthor_uid=19221208)**,** **et al.** Endurance training in the spontaneously hypertensive rat: conversion of pathological into physiological cardiac hypertrophy. [Hypertension](http://www.ncbi.nlm.nih.gov/pubmed?term=Endurance+Training+in+the+Spontaneously+Hypertensive+Rat&TransSchema=title&cmd=detailssearch) 2009; 53: 708-14.

[2] [**Dupont S**](http://www.ncbi.nlm.nih.gov/pubmed?term=Dupont S%5BAuthor%5D&cauthor=true&cauthor_uid=22287586)**,** [**Maizel J**](http://www.ncbi.nlm.nih.gov/pubmed?term=Maizel J%5BAuthor%5D&cauthor=true&cauthor_uid=22287586)**,** [**Mentaverri R**](http://www.ncbi.nlm.nih.gov/pubmed?term=Mentaverri R%5BAuthor%5D&cauthor=true&cauthor_uid=22287586)**, et al.** The onset of left ventricular diastolic dysfunction in SHR rats is not related to hypertrophy or hypertension. [Am J Physiol Heart Circ Physiol](http://www.ncbi.nlm.nih.gov/pubmed/22287586) 2012; 302: H1524-32.

[3] [**Parra V**](http://www.ncbi.nlm.nih.gov/pubmed?term=Parra V%5BAuthor%5D&cauthor=true&cauthor_uid=24009260)**,** [**Verdejo HE**](http://www.ncbi.nlm.nih.gov/pubmed?term=Verdejo HE%5BAuthor%5D&cauthor=true&cauthor_uid=24009260)**,** [**Iglewski M**](http://www.ncbi.nlm.nih.gov/pubmed?term=Iglewski M%5BAuthor%5D&cauthor=true&cauthor_uid=24009260)**,** **et al.** Insulin stimulates mitochondrial fusion and function in cardiomyocytes via the Akt-mTOR-NFκB-Opa-1 signaling pathway. [Diabetes](http://www.ncbi.nlm.nih.gov/pubmed/24009260) 2014; 63: 75-88.

[4] [**Augustus AS**](http://www.ncbi.nlm.nih.gov/pubmed?term=Augustus AS%5BAuthor%5D&cauthor=true&cauthor_uid=18552160)**,** [**Buchanan J**](http://www.ncbi.nlm.nih.gov/pubmed?term=Buchanan J%5BAuthor%5D&cauthor=true&cauthor_uid=18552160)**,** [**Addya S**](http://www.ncbi.nlm.nih.gov/pubmed?term=Addya S%5BAuthor%5D&cauthor=true&cauthor_uid=18552160)**, et al.** Substrate uptake and metabolism are preserved in hypertrophic caveolin-3 knockout hearts. [Am J Physiol Heart Circ Physiol](http://www.ncbi.nlm.nih.gov/pubmed?term=Substrate+uptake+and+metabolism+are+preserved+in+hypertrophic&TransSchema=title&cmd=detailssearch) 2008; 295: H657-66.
